# Monitoring fatigue state with heart rate‐based and subjective methods during intensified training in recreational runners

**DOI:** 10.1002/ejsc.12115

**Published:** 2024-04-26

**Authors:** Olli‐Pekka Nuuttila, Arja Uusitalo, Veli‐Pekka Kokkonen, Nilushika Weerarathna, Heikki Kyröläinen

**Affiliations:** ^1^ Faculty of Sport and Health Sciences University of Jyväskylä Jyväskylä Finland; ^2^ The UKK Institute for Health Promotion Research Tampere Finland; ^3^ Department of Sports and Exercise Medicine, Clinicum University of Helsinki Helsinki Finland; ^4^ Helsinki Clinic for Sports and Exercise Medicine Foundation for Sports and Exercise Medicine Helsinki Finland

**Keywords:** fatigue, overtraining, performance, physiology, recovery

## Abstract

The purpose of this study was firstly to examine the sensitivity of heart rate (HR)‐based and subjective monitoring markers to intensified endurance training; and secondly, to investigate the validity of these markers to distinguish individuals in different fatigue states. A total of 24 recreational runners performed a 3‐week baseline period, a 2‐week overload period, and a 1‐week recovery period. Performance was assessed before and after each period with a 3000m running test. Recovery was monitored with daily orthostatic tests, nocturnal HR recordings, questionnaires, and exercise data. The participants were divided into subgroups (overreached/OR, *n* = 8; responders/RESP, *n* = 12) based on the changes in performance and subjective recovery. The responses to the second week of the overload period were compared between the subgroups. RESP improved their baseline 3000 m time (*p* < 0.001) after the overload period (−2.5 ± 1.0%), and the change differed (*p* < 0.001) from OR (0.6 ± 1.2%). The changes in nocturnal HR (OR 3.2 ± 3.1%; RESP −2.8 ± 3.7%, *p* = 0.002) and HR variability (OR −0.7 ± 1.8%; RESP 2.1 ± 1.6%, *p* = 0.011) differed between the subgroups. In addition, the decrease in subjective readiness to train (*p* = 0.009) and increase in soreness of the legs (*p* = 0.04) were greater in OR compared to RESP. Nocturnal HR, readiness to train, and exercise‐derived HR‐running power index had ≥85% positive and negative predictive values in the discrimination between OR and RESP individuals. In conclusion, exercise tolerance can vary substantially in recreational runners. The results supported the usefulness of nocturnal HR and subjective recovery assessments in recognizing fatigue states.

## Highlights

- Tolerance to similarly increased training load varies in recreational runners. While some individuals respond positively, for others the same load may be excessive.
- Subjective markers, such as readiness to train and soreness of the legs, were the most sensitive markers to respond to increased training load, and the magnitude of these responses was greater in individuals with suspected overreaching.
- Resting or exercise heart rate (HR) did not respond as systematically as the subjective markers to the training load itself. However, the changes in nocturnal HR, nocturnal HRV, and HR‐running power index started to differ between the RESP and OR subgroups at an earlier stage of the overload period supporting their usefulness as indicators of the fatigue state.

## 1 INTRODUCTION

Classic mathematical training models assume that once an individual is exposed to a certain exercise dose, it results in positive responses in fitness, which can be realized after the alleviation of fatigue (Taha et al., 2003). The state of fatigue is typically considered as a continuum, and the level of fatigue is defined based on changes in performance and the time needed to recover (Bellinger, 2020; Meeusen et al., 2013). For diagnostic purposes, perceived recovery or mood is often evaluated along with the performance (Aubry et al., 2015; Bellinger, 2020; Coates, Hammond, & Burr, 2018). A state in which the perceived fatigue might be increased above normal, but performance is unchanged or improved is called acute fatigue, and it occurs basically after each training session but fades within day(s). If incomplete recovery accumulates long enough, the next stage of fatigue is regarded as overreaching (OR), which in the case of functional OR (FOR) takes from a few days to a few weeks to recover, but during nonfunctional OR (NFOR) the recovery period is substantially longer (i.e., weeks‐to‐months). (Meeusen et al., 2013).

Although some studies have found significant improvements after the recovery period in FOR athletes (Bellenger et al., 2016; Le Meur et al., 2013), the benefits of prescribed periods of FOR have been questioned when compared to training that induces brief periods of acute fatigue only (Bellinger, 2020; Bellinger et al., 2020). Lately, FOR has also been connected to several unfavorable physiological responses, such as increased resting arterial stiffness (Coates, Millar, & Burr, 2018), mitochondrial functional impairment (Flockhart et al., 2022), decreased glucose tolerance (Flockhart et al., 2022), and higher prevalence of upper respiratory tract infections (Hausswirth et al., 2014). Therefore, markers that are sensitive and specific to the prediction of OR could be useful by supporting the prescription of sustainable training programs.

The evolution of wearable technology has provided a lot of alternatives for the monitoring of training and recovery. Among endurance training, the most typical technologies are related to heart rate (HR) recordings at rest or during exercise. Although many studies support the usefulness of HR‐based markers (Buchheit, 2014; Düking et al., 2021; Nuuttila et al., 2022a), research evidence is somewhat conflicting regarding their accuracy in the identification of fatigue states. According to reviews by Bosquet et al. (2008) and Roete et al. (2021), HR measurements during rest or exercise would not appear to be sufficient in the detection of FOR, individually. In line with that, Bellenger et al. (2016) have also suggested that responses in the HR‐based markers should be contextualized with perceived recovery as similar responses (decrease in HR and increase in HRV) can be associated with positive training adaptations and the state of FOR. In general, subjective markers have been found to be more sensitive than objective measures in responding to changes in training load (Saw et al., 2016). However, it is critical to acknowledge that not just the sensitivity to a certain (external) training load is important. Even more relevant would be the specificity of the marker to distinguish excessive load from sustainable load for an individual, as the homeostatic stress induced by a certain training load and ability to recover is highly variable between individuals (Mann et al., 2014).

The conflicting results regarding the ability of HR‐based markers to identify fatigue states could, at least partly, relate to the methodology. For example, studies examining resting HR/HRV have mostly focused on morning recordings (Roete et al., 2021), while the usefulness of day‐to‐day nocturnal recordings remains rather unknown. Moreover, the assessment of a reliable baseline is of importance in HR‐based markers, and instead of single isolated results, longer averaging periods might be necessary (Manresa‐Rocamora et al., 2021). Some studies have induced so called “parasympathetic hyperactivity” at the state of FOR (Bellenger et al., 2016; Le Meur et al., 2013), meaning decreased HR and increased HRV along with impaired performance. However, it is inconclusive how conditional the phenomenon is to methodologies (recording condition), training execution (frequency, intensity, and volume), and background of the participants (well‐trained vs. recreational). Furthermore, as caloric restriction (Mathisen et al., 2020) and relative energy deficiency (Stein et al., 2012) have been associated with increased parasympathetic activity, low energy availability could explain some of the contradictories observed during periods of excessively high training loads.

The purpose of this study was to examine the sensitivity of HR‐based and subjective monitoring markers to intensified endurance training. Furthermore, the capability of these markers to differentiate between individuals of diverse fatigue states and the optimal cut‐off values to classify individuals correctly were investigated.

## 2 MATERIALS AND METHODS

### 2.1 Participants

A total of 32 (18 males and 14 females) recreational runners were recruited to participate in the study. The participants were healthy, aged 20–45 (men) or 20–50 (women), and trained regularly by running. The health status of all individuals willing to participate was screened via a questionnaire to exclude any diseases or regular medications that could have affected the participation. In addition, their resting electrocardiography was recorded and approved by a physician before final acceptance. From the 32 participants that were screened, there were 8 dropouts. The reasons for the dropouts were challenges with the timetable (*n* = 2), health issues unrelated to the study (*n* = 2), and injuries/soreness of the legs (*n* = 4). The baseline characteristics of the participants who performed the whole study period and attended all the tests are presented in Table 1. All the participants gave their written consent to participate, and the study protocol was approved by the ethics committee of the University of Jyväskylä.

**TABLE 1:** Baseline characteristics of the participants.

	Females (*n* = 10)	Males (*n* = 14)	All (*n* = 24)
Age (yrs.)	39 ± 8	39 ± 5	39 ± 6
Height (cm)	165 ± 8	179 ± 7	173 ± 10
Body mass (kg)	62.6 ± 8.5	80.0 ± 12.7	72.8 ± 14.0
Fat (%)	24.7 ± 5.2	14.7 ± 3.8	18.8 ± 6.7
VO_2max_ (ml/kg/min)	43.1 ± 3.4	51.2 ± 8.3	47.8 ± 7.8

### 2.2 Study protocol

**FIGURE 1:**
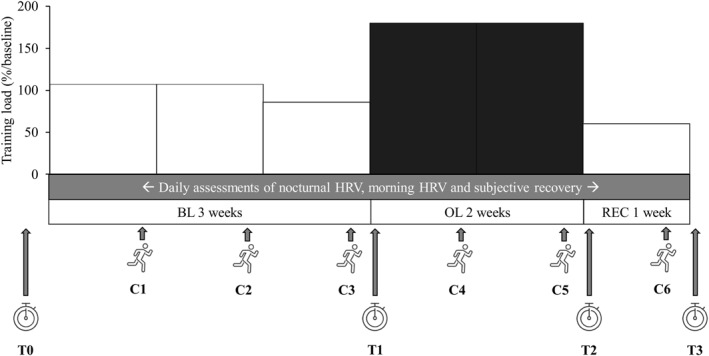
Study design. T0–T3 refers to test days, when the 3000 m running test and reactivity jump test were performed. C1–C6 refers to control running tests which consisted of a submaximal running test, countermovement jump test, and 6x3‐min high‐intensity interval training session. During the baseline period, training load of the 3rd week was slightly decreased (−20%) to ensure sufficient recovery before the overload period.

The study consisted of three distinct phases: a 3‐week baseline training period (BL), a 2‐week overload period (OL), and a 1‐week recovery period (REC) (Figure 1). Each period was followed by a test day (T1–T3) during which maximal endurance performance was assessed with a 3000 m running test. In addition, familiarization tests (T0) and a maximal incremental treadmill test were performed before the BL. Training and recovery were monitored throughout the study period by morning orthostatic tests and night HR and HRV recordings, subjective recovery questionnaires, exercise data, and weekly control running tests (C1–C6).

### 2.3 Training protocol

The training program was designed based on previous training interventions aiming to induce a momentary overload (Bellenger et al., 2016; Bourdillon et al., 2018; Dupuy et al., 2013; Le Meur et al., 2013). Furthermore, the aim was to induce overreaching in some individuals but also allow some individuals to respond positively despite comparable increase in their training load. During BL, the participants exercised at their regular frequency and volume (4–6 sessions per week). The intensity of the training was limited to low intensity (HR < first lactate threshold), except for the weekly control exercise which consisted of a self‐paced warm‐up and 6x3‐min maximum sustainable effort intervals. The intensity restriction was intended to ensure sufficient training readiness for OL.

During the OL, the training load was increased via both the intensity and the volume of training. The planned increase in the training load was 80% compared to BL as determined by the formula of Lucia et al. (2003) where the minutes spent at different intensity zones (zone 1 = 1, Zone 2 = 2, and Zone 3 = 3) have their own weight coefficients. An increase of training volume was standardized to 45%. The weekly training program during the OL consisted of a control session, 3–4 moderate‐intensity sessions (HR between lactate thresholds), and 1–2 low‐intensity sessions. The moderate‐intensity sessions were 6‐ and 12‐min intervals with a 3‐min active recovery or continuous >30 min sessions. All the moderate‐intensity sessions included a 15–20‐min warm‐up and cool‐down. The training frequency and the total volume of each training session were defined according to the training during the BL and the above‐mentioned criteria. After the OL, the training load as Lucia's TRIMP was decreased during the REC by 40% compared to BL. Except for the control session, the training was performed as low‐intensity training.

### 2.4 Performance tests

*An incremental treadmill test* was performed before the start of BL to determine individual HR zones. During the same visit, the participant's fat percentage was estimated with skinfold measurements (Durnin et al., 1967). The treadmill test started at 7 km/h (females) or 8 km/h (males), after which the treadmill speed was increased by 1 km/h every 3 min, and the test continued until volitional exhaustion. The incline was kept constant at 0.5°. The treadmill was stopped between each stage for drawing blood samples from the fingertip for lactate analyses (Biosen S_line Lab + lactate analyzer, EKF Diagnostic). The HR (Polar H10, Polar Electro Oy) and respiratory gases (Jaeger Vyntus CPX, CareFusion Germany 234 GmbH) were also measured continuously during the test. The maximal oxygen uptake (V̇O_2max_) was defined as the highest 60‐s average of oxygen uptake and the maximum HR as the highest observed value during the test. The lactate thresholds (LT1 and LT2) were defined according to the criteria used in previous studies (Nuuttila et al., 2022a, 2022b; Vesterinen et al., 2017).

T0–T3 tests (reactivity jumps + 3000 m) took place always at the same time of the day within‐individual (±2 h), and they were always preceded by a rest day. Participants were asked to follow similar nutritional habits on all test days. Since it is known that low energy availability can partly contribute to overreaching (Kettunen et al., 2021; Kuikman et al., 2022), the participants were advised to pay attention to sufficient energy intake during OL. Moreover, they received a calculation of the approximate increase in the daily energy expenditure that was caused by the increased training load. Body mass that was measured on a scale before each test was used as an indirect measure to estimate if there had been individuals with significant energy deficiency.

*Reactivity jump (RJ power) tests* were performed after a 3‐min warm up and two submaximal sets of ∼10 jumps. In the test, the participants performed 10 repeated jumps explosively with minimal contact time. Hands were held on hips and minimal bending on the knees was allowed. Contact times and flight times were analyzed from a video that was recorded with slow‐motion camera (240 fps) from the front and analyzed with MyJump Lab (Haynes et al., 2019). The power of each jump was calculated based on the formula of Bosco et al. (1983). The average of five best jumps was used in the analyses. The inter and intraday reliability of the 10/5 repeated jump test protocol has been reported in the athletic population (Southey et al., 2023).

3000 m *running tests* were conducted in small groups (max. Six persons) in a 200‐m indoor track (*n* = 18) or in a 400‐m outdoor track (*n* = 6) after the reactivity jump test. The outdoor track was used for some participants (in all tests) due to the summer lockdown of the indoor track that was not known when the timetable of the data collection was designed. A standardized 15‐min low‐intensity warm‐up including 3 × 20–30‐s accelerations to the target speed was always performed before the test. Verbal encouragement and split times were given for all participants. The running time, average and peak HR, and blood lactate concentration (post 2 min) were analyzed for each test. The 3000 m running test was chosen as an indicator of endurance performance due to its feasibility, previously reported as low coefficient of variation (CV) (1.41%) in recreational runners (Nuuttila et al., 2022b) and strong associations with incremental treadmill test variables (Nuuttila et al., 2023).

### 2.5 Training and recovery monitoring

The participants used an HR monitor (Vantage V2, Polar Electro Oy) and HR strap (H10) in all training sessions. The time in HR zones (1 < LT1, 2 = LT1‐LT2, and 3 > LT2) and the distance covered were analyzed for all the sessions. In addition, the average HR and running power of whole sessions (continuous low‐intensity) or warm‐up (moderate‐intensity) were analyzed to calculate a similar variable as the HR‐running speed index (Vesterinen et al., 2014) by replacing the running speed with running power in the formula. The HR‐running power index basically demonstrates the difference between theoretical and measured running power at a certain HR based on the individual's standing HR (BL average), maximum HR, and maximum speed of the incremental treadmill test converted to running power in the Polar Flow software. The running power was used instead of running speed to allow a fairer comparison between possibly differing terrains. An example of HR‐running power index calculation is demonstrated in the Supporting Information S1.

*Nocturnal HR recordings* were performed with validated (Nuuttila et al., 2021) photoplethysmography‐based wrist measurement (Polar Vantage V2). The results (HR, LnRMSSD) were analyzed separately for the full night and a 4‐h period which started 30 min after the detected sleep onset (Nuuttila et al., 2022c) that was based on an automatic algorithm (Pesonen et al., 2018). The results were first synced from the watch to Polar Flow software, after which consecutive 5‐min averages of the nocturnal recording data were automatically transformed to Coach4Pro software (Coach4Pro Oy, Espoo, Finland).

In the *orthostatic test*, the participants collected RR‐interval data for 2 min in the supine and standing positions. Before the measurement, the participants were instructed to empty their urinary bladder and after that go back to bed and wait for approximately 1 min before starting the recording. The data was collected by using an ECG‐based HR‐sensor (Polar H10) and the “orthostatic test” feature of the watch (Polar Vantage V2). The results were analyzed for mean HR and LnRMSSD during the second minute of the supine and standing positions.

*The control running test* consisted of a rating of a perceived exertion (RPE)‐based submaximal running test, countermovement jumps (CMJs), and a 6x3‐min maximal sustainable effort interval exercise. The test was instructed to be performed once a week in the same or similar environment and at the same time of day (±2 h) within an individual. The RPE‐based test was developed from the protocols of Sangan et al. (2021) and Vesterinen et al. (2017). It involved two 6‐min stages and one 3‐min stage with intensities defined on the RPE scale as 9 (very light), 13 (somewhat hard), and 17 (very hard). The average running speed and the average HR were calculated separately for each stage (excluding the first minute of each stage). The CMJ test (Polar Leg Recovery Test) consisting of three maximal attempts was performed before interval exercise. The jump height was estimated automatically via the inertial measurement unit in the watch (Polar Vantage V2). The validity and reliability of the method have been reported by Gruber et al. (2022). After the CMJ test, the participants performed a 6x3‐min/2‐min recovery interval session at the maximum sustainable effort. The average running speed and the average HR were determined as the average of all intervals. The average speed of the intervals has previously correlated strongly with the 3000 m running performance and its change (Nuuttila et al., 2023).

*Subjective recovery* was assessed each morning after the orthostatic test via the Coach4Pro application. The variables estimated on a 1–5 scale were as follows: sleep quality, general fatigue, stress level, readiness to train, soreness of the legs, and fatigue of the leg muscles. The scale logic was adapted from Hooper et al. (1995): Number three indicated normal perception, while increase indicated slightly or much worse perception and decrease indicated slightly or much better perception. The participants were advised to define normal the same way it was estimated at the beginning of the study and not to update, for example, possibly increasing fatigue during the study period as the “new normal”. In addition, training‐specific questions were asked in the same questionnaire consisting of session RPE on a 0–10 scale (Foster et al., 1996) and exercise perception (asking on a 1–5 scale) if previous session felt as expected or better or worse than expected. To ease the interpretation of the results, they were rescaled for the analysis as follows: Normal was defined as 0 in all the questions, decrease was defined as a negative number down to −2, and increase as a positive number up to 2 regardless of whether the change should be considered as negative or positive in perception (e.g., slightly increased soreness of the legs = 1 vs. slightly improved readiness to train = 1).

### 2.6 Classification of fatigue state

The classification of the fatigue state was based on the changes in performance and subjective recovery. Typically, the decrement in performance followed by a rebound, at least, to the baseline level is used as a criterion for FOR (Bellinger, 2020; Meeusen et al., 2013). However, as Coates, Hammond, & Burr, 2018 have argued, in recreationally trained individuals this criterion may not be suitable during overload periods due to large increases in performance, which can “mask” the subsequent performance decrements. Therefore, the fatigue state was diagnosed based on the formula proposed by Coates, Hammond, & Burr, 2018 but modified to current test protocol as follows: 3000 m time (Δ%) [(T1–T2) + (T3–T2)]. The formula combines the decrease in running performance expected from T1 to T2 with the super‐compensation expected at T3. If the change derived from the formula was greater than the smallest worthwhile change (0.3 × CV of T0–T1) (Hopkins, 2004) and the subjective readiness to train was decreased compared to BL (ten Haaf et al., 2017), the individual was defined as overreached (OR). A total of eight participants (4 females and 4 males) fulfilled this criterion. To compare the OR subgroup with individuals who completed similar increment in training load during OL but improved their performance from T1 more than the smallest worthwhile change at T2 and T3, a subgroup of responders (RESP) was formed. A total of 12 participants (3 females and 9 males) fulfilled this criterion leaving four participants who could not be classified as OR or RESP out of the subgroup analysis. One of these participants fulfilled the performance criterion for OR, but the subjective readiness to train improved significantly from BL to OL. Two participants fulfilled performance criterion for RESP at T2 but not at T3 due to impaired performance. One participant did not improve performance more than SWC at T2 but did not have supercompensation at T3 either. Instead of classifying unclear participants on case‐by‐case basis as OR or RESP, it was considered more appropriate to stick to objective, reproducible criteria that would allow more accurate comparison between individuals who in practical terms had meaningfully differing fatigue states.

### 2.7 Statistical analyses

The results are presented as mean ± standard deviation. The normality of the data was analyzed with Shapiro–Wilk test. The effects of the OL and REC periods were analyzed by repeated measures ANOVA with Bonferroni post hoc test or if the variables were not normally distributed (training characteristics) by Friedman nonparametric tests. For the performance tests, comparisons were performed between the T1–T3 time points, and for the monitoring variables, the weekly averages during the OL (OL1 and OL2) and REC periods were compared to the average of BL. Weekly averages have also been used in previous overload studies (Bellenger et al., 2016; Bourdillon et al., 2018; Le Meur et al., 2013), and they been suggested to be methodologically superior compared to single‐day results in the detection of FOR (Manresa‐Rocamora et al., 2021). Between‐subgroup (OR vs. RESP) differences in the changes from T1 or BL were examined with simple contrasts or in nonparametric tests with a Mann–Whitney *U*‐test. In addition, a receiver operating characteristic (ROC) analysis (Hajian‐Tilaki, 2013) was performed to examine how well each weekly measured monitoring marker was able to classify the participant negatively or positively as overreached. For the markers that had significant (*p* < 0.05) area under the ROC curve (AUC), an optimum cut‐off value was analyzed based on the maximum Kolmogorov–Smirnov metric. Finally, using this cut‐off value, positive and negative predictive values were analyzed. In the results of resting HR and HRV, one participant (in OR subgroup) was excluded from the analyses due to abnormally high HRV that prevented obtaining data from the watch recordings during the study period. Statistical analyses were performed with IBM SPSS Statistics v.28 program (SPSS Inc).

## 3 RESULTS

### 3.1 Training

The mean weekly training characteristics of the total group of participants and subgroups during each period are presented in Table 2. The weekly training volume increased from BL during OL 44 ± 5% (*p* < 0.001). In turn, Lucia's TRIMP increased by 73 ± 11% (*p* < 0.001) and session RPE by 122 ± 44% (*p* < 0.001). No differences were found between the subgroups regarding any training characteristics during the OL (*p* > 0.157).

**TABLE 2:** Weekly mean training characteristics during the baseline (BL), overload (OL), and recovery (REC) periods.

	ALL (*n* = 24)	RESP (*n* = 12)	OR (*n* = 8)
Volume (h)			
BL	4.5 ± 1.0	4.4 ± 0.9	4.9 ± 1.3
OL	6.5 ± 1.3***	6.4 ± 1.2***	7.0 ± 1.7***
REC	2.7 ± 0.8**	2.6 ± 0.8***	2.9 ± 0.9***
Frequency (sessions/wk)			
BL	4.3 ± 0.7	4.3 ± 0.7	4.7 ± 0.8
OL	5.4 ± 0.7***	5.4 ± 0.6***	5.6 ± 0.7***
REC	3.4 ± 0.6**	3.3 ± 0.5***	3.5 ± 0.7***
Distance (km)			
BL	43 ± 14	41 ± 12	50 ± 17
OL	65 ± 20***	63 ± 18***	73 ± 23***
REC	26 ± 8**	24 ± 6***	29 ± 12***
HRz1/z2/z3 (%)			
BL	89/8/4	88/8/3	91/6/3
OL	68/26/6***	67/27/5***	76/21/4***
REC	85/11/5*	83/12/4	88/8/4
Lucia's TRIMP			
BL	310 ± 58	304 ± 50	328 ± 77
OL	533 ± 96***	528 ± 95***	535 ± 119***
REC	193 ± 51**	192 ± 61**	198 ± 49***
Session RPE			
BL	1034 ± 206	1066 ± 185	1075 ± 2227
OL	2297 ± 623***	2356 ± 584***	2301 ± 623***
REC	711 ± 362***	787 ± 484***	666 ± 151***

*Note*: RESP = responders and OR = individuals with suspected overreaching. ***p < 0.001, **p < 0.01, and *p < 0.05 compared to BL.

### 3.2 Running and jumping performance

**FIGURE 2:**
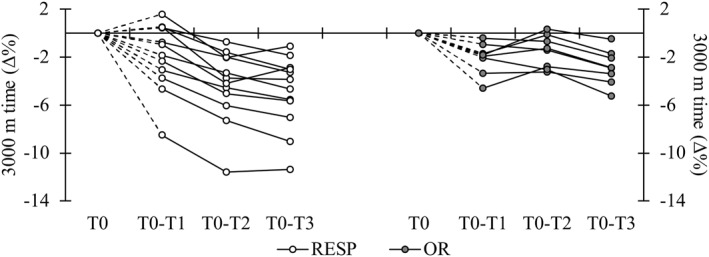
Individual responses within the subgroups. White plots refer to individual “responders” (RESP) and gray plots “overreached” (OR) individuals.

T1 3000‐m time (*p* = 0.610) or RJ power (*p* = 0.910) did not differ between the subgroups. The 3000‐m time decreased significantly in the total group of participants from T1 to T2 (−1.2 ± 1.7%, *p* = 0.006) and T1 to T3 (−1.7 ± 1.2%, *p* = 0.002). The changes from T1 to T2 (OR 0.6 ± 1.2%; RESP −2.5 ± 1.0%) and T1 to T3 (OR ‐0.8 ± 1.6%; RESP −3.1 ± 0.8%) differed between the subgroups (*p* < 0.001), and the running time remained unchanged in OR at T1–T3. The blood lactate decreased from T1 to T2 only in the OR group (*p* = 0.03), and the change differed from the RESP group (OR −2.0 ± 2.4 mmol/L; RESP 0.3 ± 1.6 mmol/L, *p* = 0.02). In HRavg or HRpeak, there were no significant changes in neither of the subgroups, but tendencies toward decrement from T1 to T2 in HRavg (*p* = 0.07) and HRpeak (*p* = 0.06) were found in the OR. The RJ power did not change in time, and no difference between the subgroups was observed. The absolute running and jumping performance test results are presented in the Supporting Information S2, and individual responses in 3000‐m within the subgroups are presented in Figure 2. The body mass remained unchanged through the study period in the total group of participants and in both subgroups.

### 3.3 Heart rate and heart rate variability in the orthostatic test and during sleep

**FIGURE 3:**
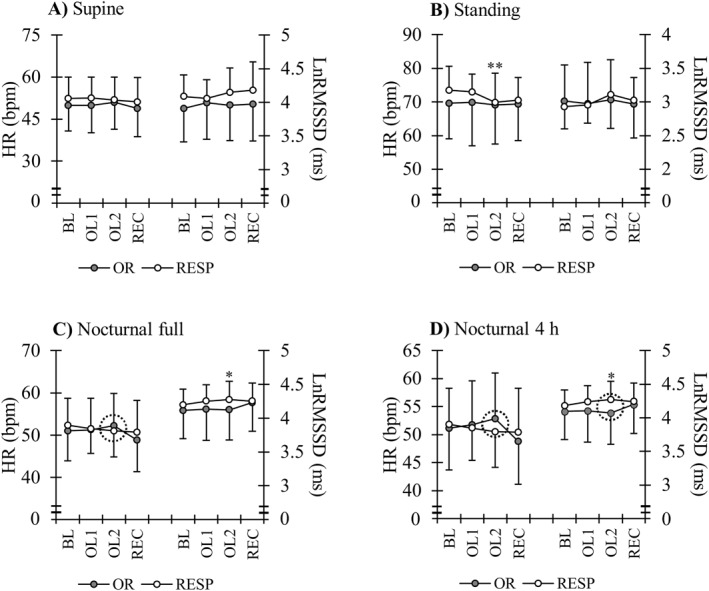
Heart rate (HR) and HR variability (LnRMSSD) in the orthostatic test (A, B) and during sleep (C, D) in the responders (RESP) and individuals with suspected overreaching (OR). BL = baseline training period; OL = overload training period; REC = recovery period. Dashed circle indicates significant between‐group difference in the change from BL. **p < 0.01 and *p < 0.05 compared to the baseline (BL) values.

The standing HR decreased from BL to OL2 (*p* = 0.02), while the nocturnal 4‐h HR decreased only from BL to REC (*p* = 0.02) in the total group of participants. In addition, the nocturnal 4‐h HRV increased from BL to OL2 (*p* = 0.04) and tended to remain increased at REC (*p* = 0.054). Subgroup analyses are presented in Figure 3. There was a decrease from BL to OL2 in the standing HR of RESP (*p* = 0.005), and the change tended to be different compared to OR (*p* = 0.054). In addition, the nocturnal 4‐h HRV increased in RESP from BL to OL2 (*p* = 0.01), and the change differed compared to OR (*p* = 0.011). The nocturnal 4‐h HR did not change within the groups, but the between‐group change from BL to OL2 was different (*p* = 0.002).

### 3.4 Changes in submaximal running performance, control running test, and CMJ

The HR‐running power index increased in the total group of participants from BL to OL2 (*p* < 0.001) and REC (*p* = 0.001). In the subgroup analyses, HR‐running power index increased from BL to OL2 and REC only in RESP (*p* < 0.001), and the change differed from OR at OL2 (*p* = 0.052) and REC (*p* = 0.009).

**FIGURE 4:**
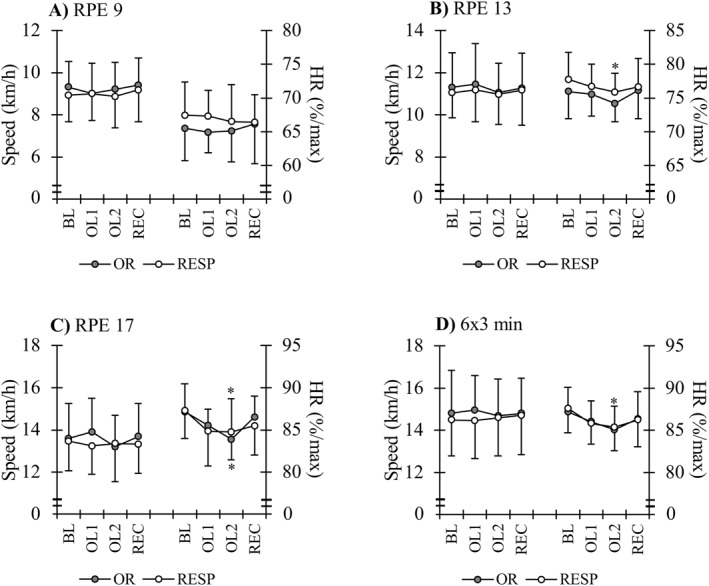
Speed and relative heart rate (HR) during the rating of perceived exertion (RPE)‐based submaximal running test and the following 6x3‐min interval session which was performed at maximal sustainable effort. BL = baseline training period; OL = overload training period; REC = recovery period; RESP = responders; and OR = individuals with suspected overreaching. *p < 0.05 compared to the baseline (BL) values.

The HR during the control running test decreased from BL to OL2 at RPE 13 (*p* = 0.001), RPE 17 (*p* < 0.001), and during 6x3‐min intervals (*p* < 0.001) in the total group of participants. The speed remained unchanged at all RPE levels and during the 6x3‐min intervals across the study period. There were no significant differences between the subgroups in any of the control test parameters (Figure 4).

The CMJ that was assessed within the control sessions decreased from BL to OL1 (−5.9 ± 7.9%, *p* < 0.01) and OL2 (−7.7 ± 8.7%, *p* = 0.003) in the total group of participants. The decrease from BL to OL2 was not significant in OR (−9.7 ± 11.0%, *p* = 0.105) or RESP (−7.4 ± 8.5%, *p* = 0.082).

### 3.5 Subjective recovery

**FIGURE 5:**
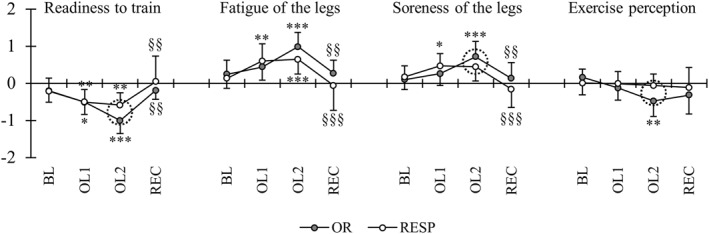
Subjective recovery in responders (RESP) and individuals with suspected overreaching (OR). Dashed circle indicates significant between‐group difference in the change from BL. ***p < 0.001, **p < 0.01, *p < 0.05 compared to BL; §§§*p* < 0.001, and §§*p* < 0.01 compared to previous week.

Subjective readiness to train decreased from BL to OL2 (*p* < 0.001), while soreness of the legs (*p* = 0.04) and fatigue of the leg muscles increased (*p* < 0.001) at OL2 compared to BL in the total group of participants. All subjective variables returned to baseline at REC and were not different compared to BL. The changes in subjective recovery within the subgroups are presented in Figure 5. Although subjective readiness to train decreased from BL to OL2 in OR (*p* < 0.001) and RESP (*p* = 0.004), the changes were greater in OR compared to RESP (*p* = 0.009). Similarly, the change in soreness of the legs from BL to OL2 differed between the subgroups (*p* = 0.04). The exercise perception decreased from BL to OL2 only in the OR group (*p* = 0.009) and the change differed from RESP (*p* = 0.02). Subjective sleep, fatigue, and stress remained unaffected compared to BL during OL1 and OL2 in the total group and subgroups of the participants.

### 3.6 Discrimination of OR and RESP individuals

**FIGURE 6:**
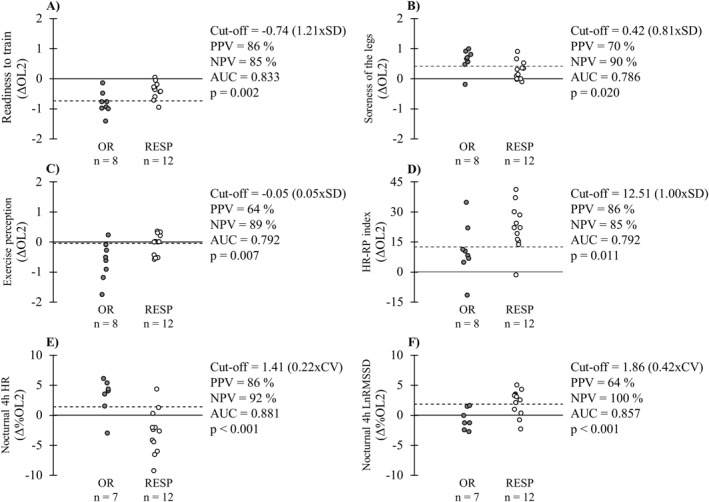
Discrimination ability of monitoring variables to correctly classify participants with suspected overreaching. Cut‐off values are presented in relation to median within individual standard deviation (SD) or CV during the baseline period. PPV = positive predictive value; NPV = negative predictive value; AUC = area under the ROC curve; RESP = responders; and OR = individuals with suspected overreaching. CV, coefficient of variation.

The variables with the most significant results in the ROC analyses are presented in Figure 6, and their 7‐day rolling average responses across the OL are provided in the Supporting Information S3. In addition to the variables presented in the figures, the AUC of supine HR (0.821, cut‐off 0.80%, *p* = 0.006), nocturnal full HR (0.845, cut‐off 0.68%, *p* = 0.001), and nocturnal full LnRMSSD (0.738, cut‐off 2.23%, *p* = 0.038) were significant.

When OL‐induced responses of monitoring variables were compared between the subgroups, changes in HR‐running power index started to differ after 6 days, in nocturnal 4‐h HR after 9 days, and in nocturnal 4‐h HRV after 10 days of OL. Among the subjective markers, changes in readiness to train started to differ on the 11th day, while soreness of the legs and exercise perception differed only on the 14th day of OL. The most valid variables to discriminate between OR and RESP individuals at the end of the OL were nocturnal HR, readiness to train, and HR‐running power index, all having ≥85% positive and negative predictive values. When these three variables were considered together and the criterion was that at least two‐thirds responses had to be on the negative side of the cut‐off limit, the positive predictive value increased to 92% and negative predictive value to 100%.

## 4 DISCUSSION

The main findings of the present study were that despite a significant increase in the training load, most of the recreational runners improved their 3000 m running performance during the 2‐week overloading period. Subjective markers, such as readiness to train and soreness of the legs, were the most sensitive markers to respond to increased training load, and the magnitude of these responses was greater in individuals with suspected overreaching. Although resting or exercise HR did not respond as systematically to the overload period itself, the changes in nocturnal HR, nocturnal HRV, and HR‐running power index started to differ between the RESP and OR subgroups at an earlier stage of the overload period, supporting their usefulness as indicators of the fatigue state.

The current training intervention (physical loading) was aimed to induce heterogenic training responses leading to functional overreaching (FOR) in ≥50% of individuals. The required increment in training load was estimated based on previous studies that have led to the state of FOR (Bellenger et al., 2016; Bourdillon et al., 2018; Dupuy et al., 2013; Le Meur et al., 2013). While it was anticipated that there would be individuals tolerating the training load well, it was rather surprising that most of the recreational runners improved their performance immediately after OL. In previous OR studies, the typical observation has been that approximately half of the participants have been diagnosed as functionally overreached (Bellinger, 2020). The decent 2‐week duration of OL was one probable reason for the lower proportion of overreached individuals. Also, the absolute training volume and the frequency of training are potential contributors. Compared to daily training during 3‐week periods (Bourdillon et al., 2018; Le Meur et al., 2013) or during a 2‐week period (Bellenger et al., 2016), the current frequency allowed 1–2 rest day(s) per week for most individuals also during OL, which seemed quite sustainable, at least in the short‐term. The potential significance of frequency is also supported by the fact that 57% (4/7) of the individuals training six or more sessions per week were in the OR subgroup, while the proportion was smaller (24%, 4/17) among individuals training five times per week. Training tolerance can also be affected by the training mode. An overload period focusing purely on running might be more challenging than cycling or combination of multiple disciplines from a musculoskeletal perspective due to high stress on muscles and tendons induced by the stretch‐shortening type of activity (Sandbakk et al., 2021). This phenomenon was prominent also in the current study as there were four individuals whose challenges with the legs, most of which started already during BL, prevented finishing the whole study period.

In line with previous research (Saw et al., 2016), subjective markers, such as readiness to train and fatigue of the leg muscles, responded quite rapidly to increased training load. These markers also had a good predictive value in the classification of the fatigue state at the end of OL supporting their usefulness when the magnitude of change is considered appropriately. Slightly surprisingly, the running speed to perceived exertion‐ratio did not respond to training load systematically and did not differ between the RESP and OR individuals. Thus, impaired subjective recovery at rest was not similarly converted to exercise performance during an overload period, although changes in effort‐based interval running performance have previously aligned with the changes in distance running performance (Nuuttila et al., 2023). As Coates, Hammond, & Burr, 2018 have discussed, especially in recreationally trained individuals, the performance itself, paradoxically, might not be an optimal marker to monitor the fatigue state, because significant improvements in fitness can mask the decrements in performance. For example, if an individual has improved his/her running time by 1.5% after the first 9 days of the overload period but decreases his/her performance by 1% during the following 5 days, the net performance remains still above the baseline level.

Among the physiological variables, the increase in HR‐running power index and decrease in exercise HR in relation to RPE‐level seemed to be the most systematic responses to the overload period as was also anticipated based on previous research (Roete et al., 2021). The weekly average of the HR‐running power index appeared more valid in the classification of fatigue state compared to control exercise performed once a week, which supports the monitoring of longer‐term trends in exercise data, similar to resting assessments (Manresa‐Rocamora et al., 2021). Interestingly, when exercise and resting HR responses were considered in relation to the fatigue state, current results contradicted many previous studies. While the systematic review of Roete et al. (2021) found that decrease in HR at fixed external load is associated with FOR and meta‐analysis by Manresa Rocamora et al. (2021) reported that weekly averages of resting HRV tend to increase in the FOR participants, the current results suggested basically the opposite. An important note among the studies included in Manresa Rocamora et al. (2021) analysis was that out of three studies that used nocturnal recordings, none reported systematically increased HRV or decreased HR in the FOR individuals. In the study of Garet et al. (2004), the results were quite similar with the present study suggesting that the decrease in parasympathetic nervous system activity during intensified training is correlated with the loss in performance. When comparing morning and nocturnal recordings, one obvious difference is in the timing in relation to preceding stressors. One hypothesis for the observed response, specifically in the 4‐h recordings, could be that OR delays the recovery of parasympathetic nervous system activity from training. Regarding the responses of exercise HR in the state of FOR, Roete et al. (2021) found decrease in relation to external load on relatively high intensities (≥80%/HRmax). The phenomenon has been suggested to relate to reduced epinephrine exertion, and the response seems to be blunted especially at higher intensities (e.g., ≥80%/peak power output) (Le Meur et al., 2014). As HR‐running power index was derived from the low‐intensity exercises, it could partly explain the differences with previous studies.

The novel approach in the current study was in the search for the optimal cut‐off values ​​for the monitoring variables to evaluate their specificity to distinguish individuals in different fatigue states. The observed patterns suggested that subjective markers, such as readiness to train and soreness of the legs, might require a larger negative change to be worthwhile (i.e., >1.0 × SD), since significant changes were observed in both groups. In turn, already a quite small negative change (e.g., nocturnal HR and exercise perception) was associated with OR in some variables suggesting a need for a narrower range (i.e., <0.3 × SD) for tolerable values. Another relevant aspect to consider in the context of monitoring is the ability to detect versus predict changes in the fatigue state. In the current study, HR‐based markers started to differ between subgroups at an earlier stage of OL than subjective markers suggesting some potential advantage of monitoring HR‐based internal responses. However, due to their own shortcomings for objective and subjective methods, the most accurate monitoring information is provided when different viewpoints are combined (Boullosa et al., 2023; Nuuttila et al., 2022a). This is also supported by the fact that the best positive and negative predictive values were achieved, when three of the most valid variables (nocturnal HR, subjective readiness to train, and HR‐running power index) were considered together.

As a certain limitation in the study, OR and/or overtraining diagnosing is still a somewhat debated topic, and it is plausible that the border lines between acute fatigue, FOR, NFOR, and overtraining are not sharp and unconditional. This was also demonstrated by four subjects whose fatigue levels could not be classified with sufficient certainty. However, the current setting most likely allowed comparing individuals who in practical terms had meaningfully differing fatigue states. The study sample was quite small, and it is important to acknowledge that the present study results, and for example, the exact cut‐off values might not be generalized outside the scope of this study. In addition, more studies are needed to examine possible sex differences in the likelihood of OR as well as OR detection methods.

### 4.1 Conclusion

In conclusion, tolerance to a certain training load can vary in recreational runners. The current results suggest that quite simple monitoring markers, such as subjective readiness to train, soreness of the legs, or exercise perception, can discriminate between differing fatigue states when the magnitude of response is considered. Furthermore, nocturnal HR and HRV recordings and the HR‐running power index derived from the exercise data may provide useful supportive information about the individual's internal response to intensified training.

## CONFLICT OF INTEREST STATEMENT

The authors declare that they have no conflicts of interest.

## Supporting information

Supporting Information S1

Supporting Information S2

Supporting Information S3
