# Supplementary material for: Monitoring fatigue state with heart rate‐based and subjective methods during intensified training in recreational runners
Source: Eur J Sport Sci. 2024 Apr 26;24(7):857–69. doi: 10.1002/ejsc.12115 (PMC11235883; doi:10.1002/ejsc.12115)
Supplement: Supplementary file 1 — Supporting Information S1 [file EJSC-24-857-s001.docx]

**SUPPLEMENT 1**

Heart rate-running speed index (HR-RS index) has originally been developed as a practical method to monitor endurance training adaptations (Vesterinen et al. 2014). The basic idea of the HR-RS index is to monitor changes in the ratio between external load (running speed) and internal load (heart rate). While the running speed is a good indicator of external load at standardized terrains, down- and uphill can alter the relationship between heart rate and running speed significantly (Lemire et al. 2018), making the between-session comparison more challenging. The current study modified the original HR-RS index and used running power (Polar white paper, 2018) instead of running speed, as it was expected to allow fairer between-session comparison regardless of the running environment. The formula for the original and modified versions of the index are provided below with an example calculation of HR-Running power index (Figure1).

**Original formula of Vesterinen et al. 2014**

HR-RS index = S_avg_ – (HR_avg_ – HR_standing_)/k

k = (HR_max_ – HR_standing_)/S_peak_

S_avg_ = average running speed (km/h) of the training session

HR_avg_ = average heart rate (bpm) of the training session

HR_standing_ = standing heart rate (bpm)

HR_max_ = maximum heart rate (bpm)

S_peak_ = Running speed (km/h) associated with the maximum heart rate

**Formula used in the current study**

HR-Running power index = Power_avg_ – (HR_avg_ – HR_standing_)/k

k = (HR_max_ – HR_standing_)/Power_peak_

Power_avg_ = average running power of the training session

HR_avg_ = average heart rate (bpm) of the training session

HR_standing_ = average standing heart rate (bpm) during the baseline period

HR_max_ = maximum heart rate (bpm) achieved in the incremental treadmill test

Power_peak_ = maximum running power (W) of the incremental treadmill test*

*Maximum running power was derived from the Polar Flow software by providing the individuals’ body mass and setting the maximum incremental treadmill test speed as the maximum aerobic power which in turn was automatically transformed to maximum aerobic power (MAP) in watts

**An example of the calculation**

Background characteristics:

Power_peak_ = 500 W

HR_standing_ = 60 bpm

HR_max_ = 200 bpm

Training session characteristics:

Power_avg_ = 380 W

HR_avg_ = 160 bpm

HR-Running power index = 380 – (160 – 60)/0.28 = 22.8

k = (200 – 60)/500 = 0.28


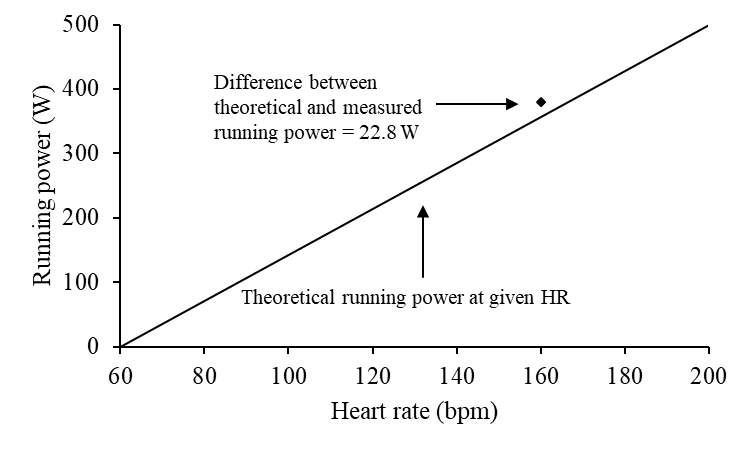


Figure 1. An example calculation of HR-Running power index with the data provided above.

**References**

Lemire, M., Falbriard, M., Aminian, K., Millet, G. P., & Meyer, F. (2021). Level, Uphill, and Downhill Running Economy Values Are Correlated Except on Steep Slopes. Frontiers in physiology, 12, 697315.

Polar Electro Oy. Polar Running Power [White Paper] 2018. [(accessed on 9 March 2024)]. Available online: [**https://www.polar.com/en/img/static/whitepapers/pdf/polar-running-power-white-paper.pdf**](https://www.polar.com/en/img/static/whitepapers/pdf/polar-running-power-white-paper.pdf)

Vesterinen V., Hokka, L., Hynynen, E., Mikkola, J., Häkkinen, K., & Nummela, A. (2014). Heart rate-running speed index may be an efficient method of monitoring endurance training adaptation. Journal of strength and conditioning research, 28(4), 902–908.
