# Supplementary material for: Monitoring fatigue state with heart rate‐based and subjective methods during intensified training in recreational runners
Source: Eur J Sport Sci. 2024 Apr 26;24(7):857–69. doi: 10.1002/ejsc.12115 (PMC11235883; doi:10.1002/ejsc.12115)
Supplement: Supplementary file 2 — Supporting Information S2 [file EJSC-24-857-s002.docx]

| **SUPPLEMENT 2.** Performance test results at the beginning of a two-week overload (OL) (T1), after OL (T2), and after a one-week recovery (REC) (T3) periods. | | | |
| --- | --- | --- | --- |
|  | **ALL**  (n = 24) | **RESP**  (n = 12) | **OR**  (n = 8) |
| **3000 m time (min:s)**  T1  T2  T3 | 12:48 ± 1:50  12:39 ± 1:48**  12:35 ± 1:53** | 12:45 ± 1:52  12:26 ± 1:50***  12:21 ± 1:50*** | 12:20 ± 1:32  12:24 ± 1:28###  12:14 ± 1:27§§§### |
| **3000 m HRavg (%/max)**  T1  T2  T3 | 92.9 ± 2.2  92.0 ± 2.4  92.6 ± 2.2 | 92.7 ± 1.8  92.2 ± 2.1  92.6 ± 2.2 | 93.6 ± 2.0  91.9 ± 2.6  92.8 ± 2.1 |
| **3000 m HRpeak (%/max)**  T1  T2  T3 | 97.9 ± 2.5  97.0 ± 2.5*  97.4 ± 2.7 | 98.0 ± 2.3  97.3 ± 2.3  97.8 ± 2.6 | 98.2 ± 2.7  96.8 ± 2.4  97.4 ± 1.9 |
| **3000 m Bla (mmol/l)**  T1  T2  T3 | 12.7 ± 3.2  12.1 ± 3.2  12.3 ± 3.3 | 12.7 ± 1.9  12.4 ± 1.8  12.4 ± 1.8 | 12.0 ± 3.9  10.0 ± 3.1*#  11.2 ± 3.5 |
| **RJ power (W/kg)**  T1  T2  T3 | 23.8 ± 7.4  23.2 ± 7.1  23.9 ± 7.2 | 24.7 ± 7.6  24.6 ± 7.8  25.4 ± 8.0 | 25.1 ± 7.4  24.1 ± 6.2  24.9 ± 8.0 |
| **Body mass (kg)**  T1  T2  T3 | 72.9 ± 13.6  72.9 ± 13.8  72.6 ± 13.6 | 77.6 ± 11.0  77.6 ± 11.2  77.2 ± 10.8 | 69.3 ± 11.0  69.7 ± 11.2  69.3 ± 10.8 |
| HRavg = average HR in relation to maximum; HRpeak = peak heart rate in relation to maximum; Bla = blood lactate concentration; RJ power = reactivity jump power; REC = recovery period; RESP = responders, OR = individuals with suspected overreaching. ***p < 0.001, **p < 0.01, p < 0.05 compared to T1; §§§p < 0.001 compared to T2; ###p < 0.001 compared to RESP | | | |
