# Supplementary material for: Monitoring fatigue state with heart rate‐based and subjective methods during intensified training in recreational runners
Source: Eur J Sport Sci. 2024 Apr 26;24(7):857–69. doi: 10.1002/ejsc.12115 (PMC11235883; doi:10.1002/ejsc.12115)
Supplement: Supplementary file 3 — Supporting Information S3 [file EJSC-24-857-s003.docx]

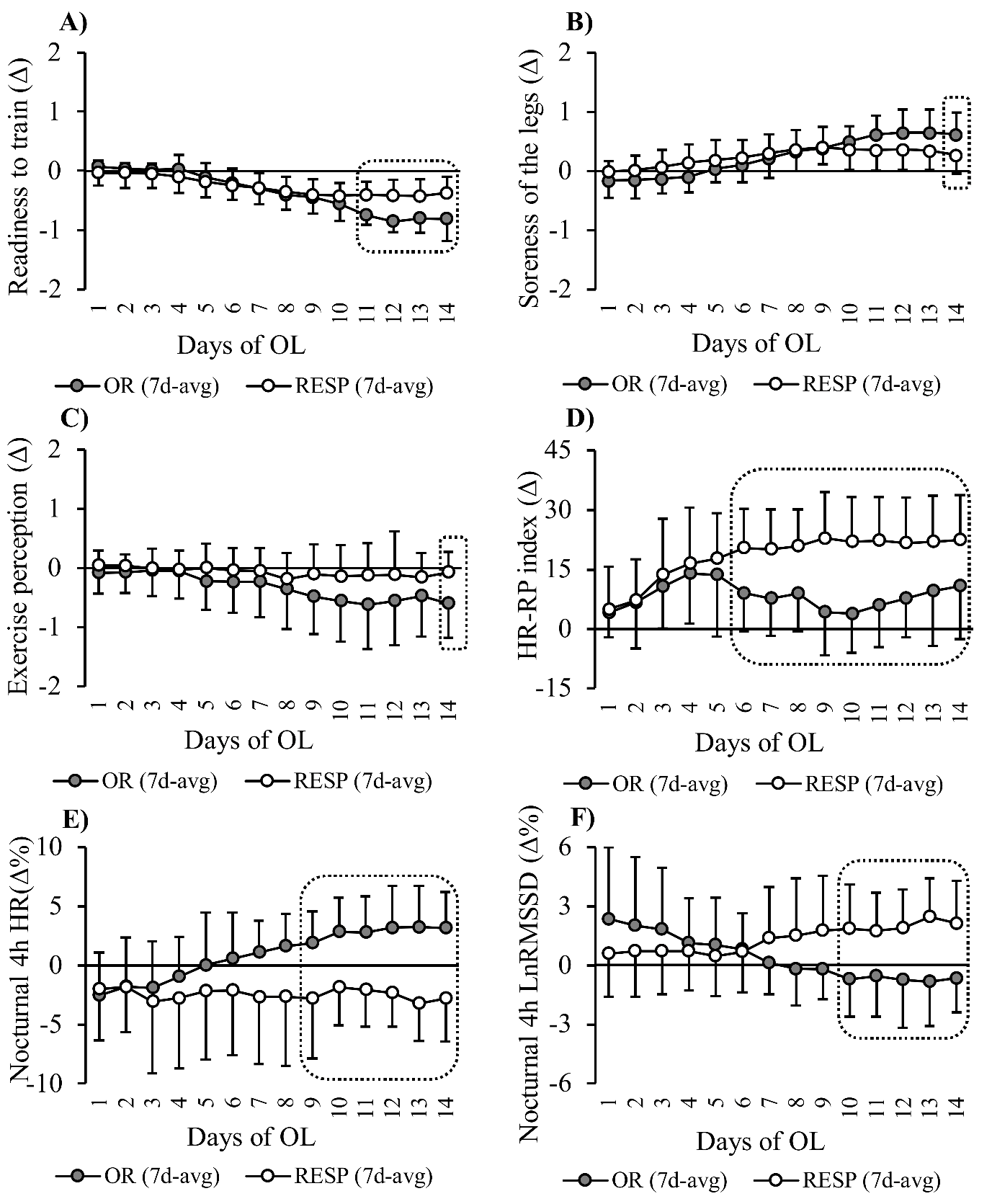


**SUPPLEMENT 3.** The variables with the most significant results in the ROC-analyses and their 7-day rolling average responses across the overload period (OL). Dashed circle indicates significant (p < 0.05) between-group difference in the change from the baseline period. RESP = responders; OR = individuals with suspected overreaching; HR-RP index = Heart rate-running power index.
